# Molar-Incisor Hypomineralization: Prevalence Comparative Study in 6 Years of Interval

**DOI:** 10.1155/2022/4743252

**Published:** 2022-12-09

**Authors:** Jéssica Damares Lago, Manuel Restrepo, Diego Girotto Bussaneli, Jéssica Patrícia Cavalheiro, Juliana Feltrin de Souza, Lourdes Santos-Pinto, Rita de Cássia Loiola Cordeiro, Fabiano Jeremias

**Affiliations:** ^1^Department of Morphology, Genetics, Orthodontics and Pediatric Dentistry, Araraquara School of Dentistry, UNESP-Universidade Estadual Paulista, Araraquara, São Paulo, Brazil; ^2^Department of Pediatric Dentistry, School of Dentistry, Universidad CES, Medellín, Antioquia, Colombia; ^3^Department of Stomatology, School of Dentistry, Universidade Federal do Paraná, Curitiba, Paraná, Brazil

## Abstract

The aim of this study was to evaluate the prevalence evolution rate of MIH (molar-incisor hypomineralization) after a 6-year period in Araraquara/SP, Brazil. This population-based study evaluated MIH in 545 schoolchildren (6–12 years of age) and other associated alterations, dental caries and dental fluorosis (DF). A semistructured questionnaire was sent to the schoolchildren's parents/guardians with the purpose of identifying the socioeconomic profile. Associations between MIH and the clinical characteristics were analyzed by the Poisson analysis of regression with robust variance, estimating the RPc (crude prevalence ratio) with CI 95%. The level of significance of 5% was adopted. The MIH prevalence in Araraquara/SP in 2016 was 14.3% (*n* = 78), and at this time, an increase of 2% was observed, in comparison with the data of the first prevalence study conducted in 2010 (12.3%). The mild degree compromise was the most prevalent diagnosis in the affected teeth (82.0%). Among children with MIH, the mean number of affected teeth was 2.78. Of the total number of children with MIH, 32.0% presented alterations in both first permanent molars and permanent incisors. There is no significant association between MIH and dental caries experience on permanent dentition (PRc = 1.141; CI 95% 0.709–1.835) or on primary dentition (PR_*c*_ = 1.132; CI 95% 0.749–1.709). Children with MIH presented significantly less prevalence of dental fluorosis (PR_*c*_ = 0.505; CI 95% 0.268–0.950). There is no association between MIH and monthly Brazilian minimal wage income (PR_*c*_ = 1.130; CI 95% 0.655–1.949). It was concluded that the number of MIH cases had increased, revealing a greater need for defining the etiological factors and establishing a correct diagnosis to make it possible to institute early intervention.

## 1. Introduction

The term “molar-incisor hypomineralization” (MIH) was suggested in 2001 to describe the clinical aspect of enamel hypomineralization that affects one or more first permanent molars (FPMs) and could be associated with permanent incisors [1]. At present, it is known that other permanent teeth may also be affected, such as the second premolars and canines, in addition to some deciduous teeth, for example, the second molars [2]. The MIH opacities have delimited margins distinct from the adjacent normal enamel. Sometimes, the porous enamel fractures easily, particularly under the masticatory forces, leaving the dentin unprotected, thereby favoring the development of caries lesions [3–7]. Furthermore, during tooth brushing and even during eruption, the teeth may be very sensitive to variations in temperature [8].

With regard to the etiology of MIH, the majority of investigations have concentrated on environmental and systemic factors that have occurred from the time of the gestational period through the first 3 years of life, a period that coincides with the mineralization stage of the first permanent molars and permanent incisors [4]. Among the most common risk factors are respiratory problems, complications in the prenatal period, low birth weight, calcium and phosphate metabolic disorders, exposure to dioxin and bisphenol A, as well as childhood diseases associated with high fever and the use of antibiotics, and vitamin deficiencies [9–12]. However, there are other factors that must also be considered when seeking the etiopathogenesis of MIH because the entire process of pathogenesis is under genetic control [13]. Some studies have pointed out evidence that genetic variations may contribute to the development of dental hypomineralization [2, 13–15].

Clinically, MIH differs from enamel hypoplasia because it is a qualitative defect, characterized by demarcated enamel opacities [4, 16]. MIH may also be distinguished from dental fluorosis, since the latter is associated with prolonged exposure to fluoride and presents diffuse opacities in enamel [16]. The differential diagnosis of pathogenesis imperfecta is based on the fact that in MIH, the molars are rarely equally compromised, whereas in pathogenesis, almost the entire dentition is affected, and there is always a correlated hereditary pattern [5].

The reported prevalence of MIH varies considerably from 2.5 to 40.2%, indicating differences in recognizing MIH, especially mild defects that are particularly hard to discern from other defects [17, 18]. Also, methodological characteristics and operational definitions of denominators and numerators to calculate prevalence proportions and incidence rates influence these epidemiological measures to some extent and thereby affect the comparability of studies [19]. Incidence rates and prevalence proportions are important to monitor diseases and inform public oral healthcare decisions. The comparison of these epidemiological measures between different populations is important, as is the investigation of factors explaining differences that lead to increased knowledge on both etiology and prevention of diseases [19].

The aim of this study was to evaluate the prevalence evolution rate of MIH in schoolchildren after a 6-year period in Araraquara, SP, Brazil.

## 2. Materials and Methods

### 2.1. Study Design and Participants

The present cross-sectional study was approved by the Research Ethics Committee of the Araraquara School of Dentistry at UNESP (CAAE-53245816.6.0000.5416). The study was reported according to the STROBE statement and applied the same methodology as the MIH study prevalence conducted by Jeremias et al., whose data were used to analyze the incidence and associated factors [20].

The sample of this study was probabilistic (population-based), considering 5% accuracy, a confidence interval of 95%, and a previous MIH prevalence of 12.3% [20]. The sample size (545 schoolchildren, 6–12 years) was divided into two groups or strata and made up of samples proportional to their sizes: group 1: schoolchildren from the private school network (*n* = 137) and group 2: schoolchildren from the public school network (*n* = 408).

With authorization from the Araraquara Secretary for Education, 54 primary schools in the city were contacted and could be visited in a period of five months. The parents signed the free and informed consent term to have their children examined in the school environment and received the questionnaire with reference to the family socioeconomic profile to verify the parents'/guardians' income and educational level, as well as information about the water consumed, based on the National Research per Sample of Dwellings.

To compose the casuistic of this study, the volunteers (schoolchildren) met the following inclusion criteria: born in Araraquara; presence of all first permanent molars (4) and permanent incisors (8), completely erupted in the oral cavity. The following criteria were reasons for noninclusion: schoolchildren with dental hypoplasia and developmental tooth enamel defects caused by amelogenesis imperfecta, children with any syndrome linked to tooth enamel malformation, children who used fixed orthodontic appliances, children with opacities present only in the permanent incisors, children whose parents did not agree with allowing them to participate in the study, and children who participated in the previous study.

### 2.2. Data Collection

The clinical exam was performed under natural light in a school environment with the help of a wooden spatula, oral mirror, and probe recommended by the World Health Organization [21]. The same examination protocol used in this study was also used in the previous study. After cleaning and drying the teeth with sterile gauze, a complete inspection was carried out by means of the following indexes: (1) presence and severity of MIH (main variable), using the EAPD (European Academy of Pediatric Dentistry) criteria [4]; severity was classified as opacities, posteruptive fractures, and atypical restorations; (2) DMFT index (decayed, missing, or filled permanent teeth) and dmft (decayed, extracted, or filled deciduous teeth) [21]; (3) no-fluoride enamel defects in primary dentition (*Modified DDE Index*) [22] and Deciduous Molar Hypomineralization (DMH) [23]; and (4) dental fluorosis [24] (both dentition). Two calibrated examiners (kappa intraexaminador: 0.94 to MIH, DF, and DMH, 0.92 to DMFT/dmft; kappa interexaminador: 0.93 to MIH, 0.92 to DMH and DF, 0.90 to DMFT/dmft) performed the exams. The calibration process was made with photographs and patients. The present study did not aim to assess the prevalence of other alterations but only to relate them to the main variable, MIH.

### 2.3. Statistical Analysis

The data were statistically analyzed using the SPSS version 20.0 software program (IBM, Rochester, MN, USA). The dependent variable was MIH; the presence of MIH was defined by the EAPD criteria as individuals with at least one first permanent molar with HMI. The presence of the clinical characteristics was computed: dental caries experience in permanent (DMFT > 1) or primary (dmft > 1) dentition; dental fluorosis as a bilateral condition with diffuse hypomineralization. The associations between MIH and other characteristics were analyzed by the prevalence ratio (PRc), calculated by Poisson logistic regression with robust variance. For all the analyses, a level of significance of 5% was adopted.

## 3. Results

A total of 1,530 children aged 6–12 years were invited, and 756 schoolchildren were examined; 545 (mean age: 8.93 ± 1.99) fulfilled the inclusion criteria and were included in the sample. The demographic data of the study population are shown in Table 1.

The MIH prevalence was 14.3% (*n* = 78), showing no significant difference with regard to gender (38 females and 40 males, *p*=0.587) and number of teeth affected (98 females and 119 males, *p*=0.545). The mean age of children with MIH was 8.5 years (±1.4) and the mean number of affected teeth per child was 2.78 ± 1.81. Schoolchildren from the private school were the most compromised by MIH (*p*=0.474).

Of the total number of children with MIH examined, 32.0% (*n* = 25) presented alterations in first permanent molars and permanent incisors; 28.2% (*n* = 22) presented hypomineralization in only one permanent molar; 38.5% (*n* = 30) in more than one permanent molar; and only 1 case (1.3%) involved the permanent molar, canine, and incisor.

A total of 217 teeth were affected by MIH, 9% (*n* = 93) were in the maxilla and 57.1% (*n* = 124) were in the mandible. The teeth most commonly affected were the permanent mandibular first molars, followed by the permanent maxillary first molars and maxillary central incisors (Table 2). In the majority of affected teeth, the severity was mild, presenting only demarcated opacities (82, 0%), whose coloring varied from white to brownish (white color being the most frequent). However, 14.2% presented posteruptive fractures and 3.8% presented atypical restorations, with a higher percentage of satisfactory clinical conditions (Table 3). In relation to affected surfaces, the occlusal surface was the most commonly affected, followed by the buccal and distal surfaces.

In the total sample (*n* = 545), DMFT was 0.47 (±1.17). For children without MIH, DMFT was 0.49 (±1.23), and with MIH, it was 0.33 (±0.75).  Table 4 presents the percentage of FPMs affected with relation to caries experience. Approximately 10.6% of the affected mandibular FPMs (*n* = 10) presented dental caries, either in their previous history (restored with/without caries) or in their present history (decayed). Similar data were noted for affected maxillary FPMs (*n* = 7; 10.0%) (*p* > 0.05). No permanent incisors showed decay.

Table 5 shows data about MIH and associated factors (clinical and demographical). Approximately 24.4% of the MIH children (*n* = 19) presented caries experience in permanent dentition (DMFT > 0), but with no relation to a higher level of dental caries experience on permanent (PR_*c*_ = 1.141; CI 95% 0.709–1.835), or on primary dentition (PR_*c*_ = 1.132; CI 95% 0.749–1.709). Children with MIH presented significantly less prevalence of dental fluorosis (PR_*c*_ = 0.505; CI 95% 0.268–0.950). Only 10 children with MIH presented DF (20.5%). No DF in the primary dentition was noted. There is no association between MIH and DMH (PR_*c*_ = 1.637; CI 95% 0.594–4.51) or with the Brazilian monthly wage income (PR_*c*_ = 1.130; CI 95% 0.655–1.949). No tooth loss by MIH was recorded.

Table 6 presents the comparative data of prevalence and severity from the two epidemiological surveys conducted in Araraquara (São Paulo/Brasil) for evaluating MIH with an interval of 6 years between them. It was possible to observe an increase in prevalence (from 12.3% to 14.8%), an increase in structural loss (5.3% to 14.2%), an increase in the number of children with defects in molars (from 48.6% to 66.7%), and reduction in the mean number of teeth with MIH and caries experience in permanent dentition (from 0.89 to 0.33). No association of MIH with fluorosis or with DMH was analyzed in study 1.

## 4. Discussion

The prevalence of MIH observed in Araraquara in 2016 was 14.3%, higher than the findings of the prevalence study previously conducted in the same city, in which a percentage of 12.3% was observed [20]. Making comparisons across prevalence studies is hard due to differences in the studied population such as age, gender, socioeconomic class, ethnic background, sample selection, and diagnosis criteria [17, 19]. The standardization of these factors in both studies allowed the results to be compared. The prevalence evolution rate of MIH was 2%. This rate indicates that there has been a change in the balance of etiological factors and that more children have been affected over the years. It is important to the researcher who intends to understand the etiology of a disease. Moreover, rates of incidence are used to make inferences about the disease risk probability [25]. As far as we are aware, there are no comparative prevalence studies on MIH performed in the same population with absolute control of the variables responsible for the variability in prevalence studies carried out around the world.

In this study, no statistical difference was observed between the genders. The equality in manifestation of the mentioned condition between the genders has also been observed in previous studies [26–28]. In the study of Jeremias et al., a higher prevalence of MIH was observed in the female gender, with girls exhibiting a 1.5 times greater chance of having MIH than boys [20].

In this investigation, the mean number of affected teeth per child by MIH was 2.70, a little below the previous study, in which a mean of 3.30 was observed [20]. The present findings were close to the mean value observed in Hong Kong (2.60) [29], but different from some European countries (2.30) [30], (5.60) [31], (5.50) [32], and (4.80) [33]. These different data may be related to several factors, including the calibration of the team to perform the diagnoses as well as the multifactorial conditions related to the manifestation of the defect among individuals. In the present study, the examiners had eight years of experience in the subject studied.

Relative to the distribution of MIH, in 28.2% of the affected children, only one affected molar was observed; 38.4% had changes in more than one molar; and 32.0% presented defects in both molars and incisors. In the Schmalfuss et al. study [34], the incisors involvement was observed in approximately 42.0% of the sample. Although the nomenclature of the enamel defects investigated suggested the characteristic involvement in permanent molars and incisors, there were reports of hypomineralization also occurring in other teeth, such as the permanent canines and premolars, and deciduous second molars, due to the coincident period of mineralization of the crown of these teeth [2, 35]. The association of some factors (genetic [16], crown formation period, and immune response [17]) can also explain these findings. According to Bussaneli et al. [36], the individual genetic load determines the intensity of the immune system's response and modulates the expression of the genes related to dental enamel maturation. Therefore, it could increase the susceptibility and severity of enamel hypomineralization in different types of teeth [37].

Mandibular molars were most affected by MIH, corroborating the findings of Jälevik et al. [3] and Hanan et al. [38]; however, the maxillary molars were the most affected teeth in Jeremias et al. [20], Muratbegovic et al. [33], and Schmalfuss et al. [34]. According to Vieira and Manton [39], this asymmetric pattern is due to differences in gene expression and environmental variations during amelogenesis.

Considering the severity of lesions, demarcated opacity was the defect most frequently observed, appearing in 82.0% of the children, a value similar to that found by Jeremias et al. [20], who observed a percentage of 90.7 in Araraquara, SP, and by Hanan et al. [38], who found a value of 84.7% in Manaus, AM, Brazil. As regards posteruptive fractures, 14.2% of the children presented teeth with posteruptive breakdown, and 3.8% presented atypical restorations, with a higher percentage of children having a satisfactory clinical condition. In the previous study, the prevalence of severe lesions was observed in 9.3%, with 5.3% presenting posteruptive fractures and 4.0% atypical restorations [20]. In the comparison between the prevalence studies conducted in Araraquara, SP, there was an increase in the percentage of structural loss, which may be related to the clinical evidence of an increase in the number of molars affected by MIH, but above all, to the structural condition and the location of the opacity on the tooth. The occlusal surface was the most commonly affected, followed by the buccal.

The teeth affected by MIH frequently present a more porous enamel, a larger accumulation of bacterial plaque, and a high level of tooth sensitivity due to the exposure of dentin, which makes these teeth more susceptible to developing caries lesions. In 2001, Weerheijm et al. pointed out that if MIH lesions were not treated, they tended to progress from a slight to severe condition over time, thus requiring more extensive interventions [1]. In this study, the children with MIH in permanent dentition presented a lower DMFT index value than the unaffected children, differing from other studies [20, 36]. Often, the diagnosis of a caries lesion can be masked by an enamel/dentin fracture associated with opacities around it. In general, in this study, an increase in cases of MIH (including fractures) and a reduction in cases of dental caries were observed.

In relation to the socioeconomic conditions, the educational level and equivalence of monthly wage income were observed to be similar for children with or without MIH. Evaluation of economic indicators is of great importance in childhood health studies, because they allow the description of this population. Family income is considered a variable with high discriminatory power; that is, children belonging to families with different income levels may present important differences in the health indicators [40]. Some studies have evaluated the interference of socioeconomic contexts in oral health of populations and not only the prevalence of caries [41]. They have demonstrated that the incidence of enamel defects (not directly MIH) may be influenced by socioeconomic conditions, as the probable result of nutritional deficiencies [42].

According to Wuollet et al. [43], MIH defects develop in early childhood, when a child's health and nutritional status may be affected by the SES of the family. We presupposed that the influence of socioeconomic factors on nutritional deficiencies can influence the development of MIH, especially due to lower income levels. Recent cross-sectional studies have observed an association between MIH and socioeconomic status (SES) [43], low maternal educational level [44], and nutritional deficiency [45]. In the present study, the instrument used for collecting information on the socioeconomic level was family income and the parents' educational level. Therefore, more in-depth conclusions on the socioeconomic context cannot be extrapolated in this study because it would be necessary to use a standard formula (more complex) with diverse questions to deduce the socioeconomic pattern of the family, as recommended by the Brazilian Institute of Geography and Statistics (IBGE).

Since this type of enamel hypomineralization was disclosed, the association with the ingestion of fluorides has been evaluated. Several studies have observed no association between exposure to fluoride and demarcated opacities [10]. Only in the study of Angelillo et al. was an increase in diffuse and demarcated opacities observed with regard to the level of water fluoridation [46]. In this study, no association was observed between DF and MIH since the majority of the children with MIH presented no DF (*n* = 68; 79.5%). It is important to state that the present study did not aim to assess the prevalence of other tooth alterations but only to relate them to the main variable, MIH.

In an effort to hypothesize the reason for the increase in MIH in the city of Araraquara-SP over the course of 6 years, we have to consider events that caused the dysfunction of ameloblasts in the maturation stage of the dental enamel matrix, thus generating the demarcated opacities characteristic of MIH. The probable multifactorial nature of this condition suggests that from the time of the prenatal period up to the first days of life, the environment acts in synergy with the genetic load; however, studies are necessary to find scientific evidence. Although not evaluated in this study, it is necessary to reflect on the change in lifestyle of persons over the course of the years, a fact observed worldwide. Society began to consume a large quantity of medications, perhaps because people acquired more diseases; the diagnosis of systemic alterations resulting from stress has become more frequent; and simultaneously, the consumption of industrialized foods has increased and so have the levels of environmental pollutants.

Although this study presents retrospective information, such information is extremely relevant to understanding the dynamics of the disease over the years. New comparative studies at the same location should be carried out around the world to assess the epidemiological behavior of this condition in different ethnic groups.

In clinical practice, recognizing that both the percentage and degree of severity of MIH have risen reinforces the need for an early approach with adequate preventive and interceptive treatment. Therefore, prevalence studies are fundamental for understanding the dimension of the disease in a certain population, especially in the context of public health. From the data obtained in the two studies, the authors concluded that the number of cases of MIH had increased, revealing a greater need for defining the etiological factors and establishing a correct diagnosis to make it possible to institute early intervention [20].

## Figures and Tables

**Table 1:** Demographic data of the study population.

Variables		*N* (%)
Gender	Female	281 (51, 6)
	Male	264 (48, 4)

Age	6 years	84 (15, 4)
	7 years	75 (13, 7)
	8 years	80 (14, 7)
	9 years	80 (14, 7)
	10 years	80 (14, 7)
	11 years	73 (13, 4)
	12 years	73 (13, 4)

Mean age (DP)	8.93 (1.99)	

Monthly family income	No income	2 (0, 5)
	Up to 1 salary (200 USD)	53 (12, 4)
	From 1 to 2 salaries (200–400 USD)	138 (32, 2)
	From 2 to 3 salaries (400–600 USD)	132 (30, 8)
	From 3 to 4 salaries (600–800 USD)	69 (16, 1)
	From 5 to 10 salaries (1.000–2.000 USD)	23 (5, 4)
	10 to 20 salaries (2.000–4.000 USD)	9 (2, 1)
	More than 20 salaries (>4.000 USD)	2 (0, 5)

Parental education level (mean)	Less than 8 years	158 (29, 0)
	Over 8 years	387 (71, 0)

DMFT	DMFT = 0	425 (78, 0)
	DMFT > 0	120 (22, 0)

dmft	dmft = 0	317 (58, 2)
	dmft > 0	228 (41, 8)

MIH	MIH = 0	467 (85, 7)
	MIH > 0	78 (14, 3)

MIH	Public school	56 (13.7)
	Private school	22 (16.2)

DMH	DMH = 0	532 (97, 6)
	DMH > 0	13 (2, 4)

DF	DFs = 0	422 (77, 4)
	DF > 0	123 (22, 6)

DMFT: decayed, missing or filled permanent teeth; dmft: decayed, missing or filled deciduous teeth; DMH: deciduous molar hypomineralization; DF: dental fluorosis.

**Table 2:** Frequency of permanent teeth affected by MIH, according to affected arch and hemiarch.

Affected tooth	Maxillary arch, *n* (%)		Total	Mandibular arch, *n* (%)		Total
	Right hemiarch	Left hemiarch		Right hemiarch	Left hemiarch	
Central incisor	13 (6.0)	5 (2.3)	18 (8.3)	4 (1.8)	10 (4.6)	14 (6.4)
Lateral incisor	2 (0.9)	1 (0.5)	3 (1.4)	4 (1.8)	9 (4.2)	13 (6.0)
First molar	38 (17.5)	32 (14.8)	70 (32.3)	40 (18.4)	54 (24.9)	94 (43.3)
Second molar	1 (0.5)	1 (0.5)	2 (0.9)	1 (0.5)	1 (0.5)	2 (0.9)
Canine	0 (0.0)	0 (0.0)	0 (0.0)	1 (0.5)	0 (0.0)	1 (0.5)

Total	54 (24.9)	39 (18.1)	93 (42.9)	50 (23.0)	74 (34.2)	124 (57.1)

**Table 3:** Frequency of teeth affected by MIH, according to observed alterations.

MIH alterations								Total, *n* (%)
Demarcated opacities, *n* (%)			Posteruptive fractures, *n* (%)			Atypical restorations, *n* (%)		
White	Yellow	Brownish	Mild	Moderate	Severe	Satisfactory	Unsatisfactory	217 (100.0)
104 (47.9)	61 (28.1)	13 (6.0)	26 (11.9)	5 (2.3)	0 (0.0)	7 (3.2)	1 (0.6)	

**Table 4:** Frequency of first permanent molars (FPM) affected or not by MIH, according to caries experience.

Dental condition	Right maxillary FPM, *n* (%)		Left maxillary FPM, *n* (%)		Right mandibular FPM, *n* (%)		Left mandibular FPM, *n* (%)	
	MIH > 0	MIH = 0	MIH > 0	MIH = 0	MIH > 0	MIH = 0	MIH > 0	MIH = 0
Sound	34 (89.5)	36 (97.3)	29 (90.7)	40 (100.0)	37 (92.5)	26 (100.0)	47 (87.1)	34 (100.0)
Decayed	3 (7.9)	1 (2.7)	1 (3.1)	0 (0.0)	2 (5.0)	0 (0.0)	3 (5.5)	0 (0.0)
Restored/decayed	1 (2.6)	0 (0.0)	1 (3.1)	0 (0.0)	0 (0.0)	0 (0.0)	3 (5.5)	0 (0.0)
Restored/caries-free	0 (0.0)	0 (0.0)	1 (3.1)	0 (0.0)	1 (2.5)	0 (0.0)	1 (1.9)	0 (0.0)

Total	38 (100.0)	37 (100.0)	32 (100.0)	40 (100.0)	40 (100.0)	26 (100.0)	54 (100.0)	34 (100.0)

**Table 5:** Association between MIH and associated factors (clinical and socioeconomical).

Variables	MIH > 0, *n* (%)	MIH = 0, *n* (%)	*P* value	PR_*c*_ (CI 95%)
Caries in the permanent dentition				
DMFT = 0	59 (75.6)	366 (78.3)	0.588	Reference 1.141 (0.709–1.835)
DMFT > 0	19 (24.4)	101 (21.7)		
Caries in the primary dentition				
dmft = 0	43 (55.1)	274 (58.6)	0.557	Reference 1.132 (0.749–1.709)
dmft > 0	35 (44.9)	193 (41.4)		
Dental fluorosis				
DF = 0	68 (79.5)	354 (75.8)	**0.034**	Reference 0.505 (0.268–0.950)
DF > 0	10 (20.5)	113 (24.2)		
Deciduous molar hypomineralization				
DMH = 0	75 (96.1)	457 (97.8)	0.361	Reference 1.637 (0.594–4.51)
DMH > 0	3 (3.9)	10 (2.2)		
Monthly family income				
>3 MFI	18 (13.4)	116 (86.6)	0.194	Reference 1.130 (0.655–1.949)
<2 MFI	29 (15.2)	162 (84.8)		

PR_*c*_: crude prevalence ratio calculated by Poisson regression with robust variance; CI 95%: confidence interval 95%; DMFT: decayed, missing or filled permanent teeth; dmft: decayed, missing, or filled deciduous teeth.

**Table 6:** Descriptive data of two prevalence studies carried out in the city of Araraquara, with an interval of 6 years.

Survey	2010	2016
Prevalence	12.3%	14.3%
Affected teeth (mean)	3.32	2.78
Teeth with opacities (%)	90.7	82.0
Teeth with structural loss (%)	5.3	14.2
Teeth with atypical restoration (%)	4.0	3.8
Children with MIH in molar (%)	48.6	66.7
Children with MIH in molar and incisor (%)	51.4	33.3
Significant association of MIH with dental caries	Permanent dentition	No
Mean DMFT in children with MIH	0.89 (±1.18)	0.33 (±0.75)
Mean dmft in children with MIH	1.24 (±1.82)	1.45 (±2.48)
Significant association of MIH and other DDEs in primary dentition	No	No

DDEs: developmental defects of the enamel.

## Data Availability

All datasets supporting the conclusions of this article are included within the article.
